# Avignon Hospital

**Published:** 1833-07

**Authors:** 


### REPORTS

### FROM HOSPITALS AND MEDICAL INSTITUTIONS.

*To the Editors of the London Medical and Physical Journal.*

GENTLEMEN: Should the following cases, which I met with in the course of my reading, illustrative particularly of the physiology of the cerebellum, be suited to your pages, I shall feel obliged by your inserting them this month.

I am, sir, your obedient servant,

W. HALEY HOLM,

*55, Norton street.*

### AVIGNON HOSPITAL.

*Satyriasis, consecutive to a Blow received on the Occipital Region.* By M. CHAUFFARD, Medicin à Avignon.

THE following account has appeared to me instructive and remarkable, at this time particularly, when the multiplicity of the organs in the cerebral mass is studied with as much delicacy as success. The occurrence took place at Avignon, a few months ago, and was related to me by the medical attendant of the man.

Charvet, fifty five years of age, of mild manners, of peaceable character, and of great devotion, fell down in his chamber, and struck the neck violently against one of the bed-posts. Puffiness of the inferior occipital region, and after singular alteration of the habits of the patient, followed. He is seized with a violent and continued satyriasis, and with such salacity, that he pursued beyond measure his wife, his daughters, and all those females who came in his way. This man, formerly so pious, so modest, fell into the most erotic delirium, and abandoned himself without measure to proposals and acts the most indecent. This state increased for about three months, during which time his mind and strength became weakened; when, following a violent passion, which was occasioned by the refusal of his wife *lassato viro et satiata,* he fell into a convulsion, and complained, in consequence, of an acute pain in front of the summit of the head, (where the religious feelings are situated,) and did not feel again that which he had experienced at the posterior inferior part of the cranium. Paralysis of the left side came on, cessation of the satyriasis, and erotic delirium; religious mania, continual muttering of prayers, &c. These phenomena continued till death, which occurred eight days after this change of the morbid symptoms.

The body was not opened. These facts being thus incomplete, I cannot draw any positive conclusion, but leave it to the reflection of intelligent physiologists and learned men, who do not deny without examination the beautiful researches of Gall and Spurzheim. For the future, for the progress of their science, it will be necessary to observe attentively the state of the encephalic mass in these cases, in the different sorts of mania and other maladies intimately connected therewith, according to the phrenological doctrine, with suffering and derangement of function of each special portion of the brain.

We shall probably find exact relations between cerebral lesion and phenomena, which have appeared lately quite independent, and which in reality have no other cause. If the violent blow received by the patient, of which I have succinctly related the history, did not produce a special alteration of the function of the cerebellum, and if the cerebellum do not preside over (the function of physical love) amativeness, how otherwise can the sudden manifestation of this extraordinary satyriasis be accounted for?

With regard to the religious delirium, which supervened a few days before death, this, according to all appearances, is the rational explanation. Following strong excitement, congestion, and probably cerebral hemorrhage, injection of the encephalic substance, exaltation of sensibility of the organs, which in the natural state was predominant, and that most frequently in exercise; injury sufficiently indicated by the coincidence of pain at the middle part, posterior and superior to the coronal, with exaggeration of the habitual religious sentiments of the patient.

All the treatment he underwent were some trifling drinks, and the application of two leeches to each temple three days before death; which application was followed by considerable subcutaneous œdema, and exasperation of the symptoms.

This cause of satyriasis, and of erotic mania, has been very accurately noticed by many other authors besides Gall and Spurzheim. "Considerable wounds in the neck, blows which have only glanced upon this part," says Bischoff, "have been followed by inflammation of the genital organs."—*Exposition de la Doctrine de Gall, traduit de l'Allemand, par Barbegniore*, page 84.

"The material condition of satyriasis resides in the encephalon; and in all cases its disorderly manifestation corresponds either with the natural strength and preponderance of the cerebellum, or to circumstances which, at the moment actually of the disease, have put it violently into action."—*Voisin, des Causes, Morales and Physique, des Maladies Mentales*, pp. 275-6.

The conclusion from these observations is, that satyriasis, priapism, inflammation of the sexual organs, not proceeding from external violence, particularly when these phenomena coexist with erotic delirium, ought not to be treated exclusively by antiphlogistics applied to the genital organs; but there should be associated with these measures treatment appropriate to the morbid activity of the cerebellum.

This has been done with success in several instances; and M. Voisin, particularly, has assembled in his work a series of cases conclusive on these various points. The strong disposition to excitement of the sexual organs in hydrocephali, when the cerebellum is, of all the parts of the brain, the least altered, has been given by Gall, and other anatomists, as a proof in favour of the opinion of the function of this part of the encephalon. I recollect having seen, about eleven years since, in Paris, a hydrocephalis, between fourteen and fifteen years of age, with a large head, who was guilty of masturbation to a great extent.

In 1833, when accompanying the prefect in the departmental circuit, for the examination of young persons set apart to be reformed, a herdsman of a robust form, with red beard and hair, of an infectious smell, took off his clothes, suffering from a disease which he dared not name. This was in November, in cold weather, and in a large and cold room. Scarcely was he stripped of his clothes, though before the penis became erect, his face reddened, eyes troubled, quite confused, turning the back to us, and unable to avoid this monstrous satyriasis. This state of priapism continued without relief after the spermatic secretion had taken place, and involuntary startings also; otherwise he had a heavy appearance, but answered reasonably.

This unfortunate fellow complained of being tormented with continual erections and seminal emissions; he had contracted the same habit as in the case above mentioned. He was cured. The neck was short, and the back part large and thick; the posterior portion of the occiput very wide, and consequently the cerebellum region of the cranium very much developed and predominating.

*Hydrocephalus, with Tubercle and Polypus in the Cerebellum, in a Child of Four Years of Age.* By Dr. Guillet, of Niort, Department des Deux Sevres.

James Philip Cardinal, a seven months' child, presented at birth a very wasted appearance, and his limbs were as though atrophied. Immediately after the birth, I remarked a projection very distinct over the fontanelles, as in cases of hernia cerebri During life this child always continued weak, puny, and the prey of a slow and continued fever. From the third and fourth month there was frequent epistaxis, progressive predominance of the head over all the other parts of the body; tardy dentition, (at thirteen months only the first incisors began to appear.) This period of life was not passed without various attacks; diarrhœa, nervous tremblings of the limbs, general convulsions, swelling of the cervical ganglions, &c. Towards the second year, in proportion as perception developed itself, it was remarked the child sought always a position to rest the head, and, when he was deprived of it, he supported it with one of his hands. In order to look to the right or left, he could not rotate the head on its axis, but was obliged to turn the whole body, as those do who are wry-necked. At two years of age the head was larger than that of an adult, the sincipital region very much flattened, and it presented laterally an enormous bulging out.

At three years, he fell down, and the head received the principal shock. From that time he uttered plaintive cries, and there was acute and continued pain in this part of the body. He had no inclination to exercise the body, nor did he mix with the children of his own age, but was taciturn and morose, and remained always either sitting or lying down. When the animal life commenced losing its activity, it was not so with organic life; for he ate and drank very well. From the time of the fall the convulsions came on much more frequently; one day, during one of the convulsions, he vomited some lumbricales.

Four months after the fall, he was suddenly struck with hemiplegia of the left side, to which he ordinarily inclined the head. The arm and leg of this side were entirely without motion, and the tongue participated in the paralysis.

In speaking, each word was interrupted by a moment of silence, as though an interval of repose was necessary for the brain. A few days after he became suddenly blind; the pupils were dilated, immoveable, and the eyes a little dull. At three years and a half, paralysis became complete in the lateral parts of the body; the right hand was the last part attacked. There was an extreme contraction of the fingers. The bladder also was paralysed, and incontinence of urine followed.

Reduced to such a deplorable state, this child remained sometimes forty-eight hours without wishing to take nourishment, nor even drink.

On coming forth from this extreme indifference for all that related to the wants of life, he gave proofs still, by some words imperfectly articulated, by signs or by cries, the desire to take food.

Five weeks before his death, the faculty of hearing, which until this time had still existed, was totally annihilated. Thus, neither motion, sight, speech, nor hearing, was left; he only uttered a faint cry. He took merely sugar and water for nourishment. Following numerous convulsions, which increased each day, came on complete immobility and tetanic stiffness. At times the child appeared to recognize me, for he could still form a confused judgment, without doubt, of the position in which he was placed: if the mother placed him on the bed, immediately he uttered plaintive cries; if she took him in her arms, immediately the cries ceased. He died September 12th, 1832, in a violent convulsion.

The post-mortem examination was made twenty-four hours after death. The rigidity of the body extreme; superior and inferior extremities strongly contracted; fingers of the left hand so bent into the palm, that the skin of this part had been excoriated by the nails; general emaciation; (thus this child had lived at the expense of his own substance for some time;) eyes dull and deeply sunk in the orbits; pupils retracted; crystallines opaque; cheek-bones very prominent; in short, the hippocratic face.

Total volume of the head enormous; its form that of a sphere flattened at the sinciput, little developed in front, and very prominent laterally.

The fontanelles extended an inch and a half in their transversal diameter. On opening the cranium, I found the dura mater become a fibro-cartilaginous tissue, whose resistence opposed the formation of hernia cerebri. When the scalpel had penetrated this membrane, there ran out about two ounces of serous limpid liquid. The blood-vessels which ramified on the cerebral hemispheres, as well as those which went to ramify in the cerebral substance, were gorged with blood. On pressing lightly the two lobes of the brain, we felt the fluctuation of a liquid poured out in the lateral ventricles. After having opened these two cavities, I made the following remarks: The cerebral substance was of its natural consistence; the ventricles, whose walls were of a white faded colour, were found enormously increased at the expense of the superior parts of the lobes of the brain; these cavities contained a serous and very clear liquid, of which the quantity amounted to fourteen ounces; the optic thalami (great posterior ganglions), corpora striata (great anterior ganglions), the cornua ammonis, and the plexus choroides, were developed in a direct ratio to the extent of these cavities.

In the left lobe of the cerebellum was found a tubercle of the volume of a pullet's egg, of a rounded form, and of a yellowish-green colour; its tissue externally hard, softened at the centre. This casual production, developed at the expense of the substance of the cerebellum, (for it replaced all the anterior part, which was positively wanting,) was immediately in contact with the meninges.

In separating the brain from the spinal cord, I found, resting on the basilar surface of the sphænoid, a polypus, of the size of a walnut, adhering to the cerebellum, and which I was able to crush between the fingers. The left portion of the cerebellum presented nothing worthy of note.

This case, which is not certainly new of its kind, appears to me to offer some interest with relation to the coincidence of the divers organic alterations which it presents, and after that of the march of the phenomena which were the result of it. Serous secretion, tubercle and polypus of the brain; such the nature of the affection: perversion, uneasiness of inervation, convulsions, progressive paralysis, and death; such were the consequences.

In many cases of cerebral apoplexy, (there even where pathological anatomy has done nothing to discover any thing appreciable to our senses,) where the patients are suddenly as it were struck with lightning, the observing physician has had reason to be astonished in seeing such subjects drag on their existence for whole years, in which there has been a series of disorders as marked as in the case before us.

It would be very difficult to conceive, if the symptoms were always in relation to the extent of lesion, that this unfortunate child had been able so long to sustain a vegetative life, in which the greater part of the sensorial organs have been successively struck as with death in detail. In waiting on this point for satisfactory explanations, we are obliged to acknowledge that in certain diseases there reigns much obscurity on the relation of the symptomatic effects with their morbid causes.

